# Expanding access to medications for opioid use disorder in primary care clinics: an evaluation of common implementation strategies and outcomes

**DOI:** 10.1186/s43058-022-00306-1

**Published:** 2022-07-06

**Authors:** Hannah Cheng, Mark P. McGovern, Hélène Chokron Garneau, Brian Hurley, Tammy Fisher, Meaghan Copeland, Daniel Almirall

**Affiliations:** 1 Center for Behavioral Health Services and Implementation Research, Division of Public Mental Health and Population Sciences, Department of Psychiatry & Behavioral Sciences, Stanford University School of Medicine, Palo Alto, CA USA; 2 Division of Primary Care and Population Health, Department of Medicine, Stanford University School of Medicine, Stanford, CA USA; 3 Los Angeles County Department of Public Health, Los Angeles, CA USA; 4 Department of Family Medicine, University of California, Los Angeles, CA USA; 5 Aledade, Inc., Bethesda, MD USA; 6 Center for Care Innovations, Oakland, CA USA; 7 Institute for Social Research, University of Michigan, Ann Arbor, MI USA; 8 Department of Statistics, University of Michigan, Ann Arbor, MI USA

**Keywords:** Implementation science, Dissemination and implementation science, Implementation strategies, Opioid use disorder, Public health

## Abstract

**Background:**

To combat the opioid epidemic in the USA, unprecedented federal funding has been directed to states and territories to expand access to prevention, overdose rescue, and medications for opioid use disorder (MOUD). Similar to other states, California rapidly allocated these funds to increase reach and adoption of MOUD in safety-net, primary care settings such as Federally Qualified Health Centers. Typical of current real-world implementation endeavors, a package of four implementation strategies was offered to all clinics. The present study examines (i) the pre-post effect of the package of strategies, (ii) whether/how this effect differed between new (start-up) versus more established (scale-up) MOUD practices, and (iii) the effect of clinic engagement with each of the four implementation strategies.

**Methods:**

Forty-one primary care clinics were offered access to four implementation strategies: (1) Enhanced Monitoring and Feedback, (2) Learning Collaboratives, (3) External Facilitation, and (4) Didactic Webinars. Using linear mixed effects models, RE-AIM guided outcomes of reach, adoption, and implementation quality were assessed at baseline and at 9 months follow-up.

**Results:**

Of the 41 clinics, 25 (61%) were at MOUD start-up and 16 (39%) were at scale-up phases. Pre-post difference was observed for the primary outcome of percent of patient prescribed MOUD (reach) (*β*_time_ = 3.99; 0.73 to 7.26; *p* = 0.02). The largest magnitude of change occurred in implementation quality (ES = 0.68; 95% CI = 0.66 to 0.70). Baseline MOUD capability moderated the change in reach (start-ups 22.60%, 95% CI = 16.05 to 29.15; scale-ups −4.63%, 95% CI = −7.87 to −1.38). Improvement in adoption and implementation quality were moderately associated with early prescriber engagement in Learning Collaboratives (adoption: ES = 0.61; 95% CI = 0.25 to 0.96; implementation quality: ES = 0.55; 95% CI = 0.41 to 0.69). Improvement in adoption was also associated with early prescriber engagement in Didactic Webinars (adoption: ES = 0.61; 95% CI = 0.20 to 1.05).

**Conclusions:**

Rather than providing an all-clinics-get-all-components package of implementation strategies, these data suggest that it may be more efficient and effective to tailor the provision of implementation strategies based on the needs of clinic. Future implementation endeavors could benefit from (i) greater precision in the provision of implementation strategies based on contextual determinants, and (ii) the inclusion of strategies targeting engagement.

**Supplementary Information:**

The online version contains supplementary material available at 10.1186/s43058-022-00306-1.

Contributions to the literature

- Copious research has been done to develop and evaluate implementation strategies—yet, much remains to be understood about the drivers of their effectiveness.
- The work presented here advances our understanding of the impact of pre-implementation capability and engagement on the effectiveness of implementation strategies.
- Findings suggest that an all-clinics-get-all-components package of implementation strategies may be both inefficient and ineffective. Tailoring implementation strategies based on contextual determinants may improve precision and maximize engagement.

## Introduction

### Background

Opioid overdose deaths in the USA continue to rise with the use of prescription opioids, heroin, synthetic opioids, and tainted stimulants. More than 100 individuals die daily from an opioid overdose [1]. Although three evidence-based medications for opioid use disorder (MOUD: buprenorphine, naltrexone, and methadone) exist and are available through the Drug Addiction Treatment Act of 2000 [2–5], only 10% of people across the country who need these medications receive them [6–8]. To combat the opioid epidemic, unprecedented federal funding has been directed to states and territories to increase access to MOUD and reduce opioid overdose deaths through prevention, treatment, and recovery efforts [9–15].

Among other efforts, California deployed an 18-month multi-component implementation program within local safety-net, primary care clinics to install or expand access to medications for opioid use disorder (reach), increase the number of active x-waivered providers (adoption), and augment MOUD quality of care (implementation quality). These primary care clinics were either at the start-up phase, with no current MOUD capability, or at the scale-up phase, with some existing MOUD capability.

Safety-net primary care clinics offer services to low-income and underserved populations regardless of their ability to pay [16]. Because they are usually the first point of contact for identifying and treating health conditions, these clinics are well-positioned to screen, triage, and treat opioid use disorder (OUD). Patients receiving care at these clinics often face adverse social determinants of health and added challenges to accessing specialty care services [17]. Clinicians at these clinics also report having higher time pressure and staff burnout [18, 19]—an important consideration when asking them to engage in implementation activities.

The multi-component implementation program combined access to four commonly used implementation strategies: (1) Enhanced Monitoring and Feedback, (2) Learning Collaboratives, (3) External Facilitation, and (4) Didactic Webinars. These were selected based on the evidence from the literature and lessons learned from a previous iteration of implementing MOUD in a similar setting [20–22]. Typical of other real-world implementation endeavors [23, 24], no plan was devised for offering different strategies to different clinics based on MOUD capability, specific implementation barriers, or other contextual factors. In large part, this is due to a dearth of literature or implementation expertise on how best to accomplish this. The present work evaluates the relative effectiveness of four commonly used strategies in implementation practice on reach, adoption, and implementation quality given determinants of context and engagement.

Despite massive economic investments, the opioid crisis is far from resolved. Findings from this study not only contribute to future efforts on implementing MOUD and optimizing system-level efforts to alter the trajectory of the opioid epidemic, but they may also be relatable to other implementation efforts within safety-net practices, primary care clinics, and/or other medication management settings to address the questions of how heterogeneous contextual determinants and engagement in implementation strategies may differentially impact the outcomes of an implementation.

## Methods

### Aims

Specific aims and hypotheses were specified prior to data analysis.

The primary aim was (a) to test whether the provision of the multi-component implementation program impacted change in the percent of patients prescribed MOUD (primary outcome); (b) to estimate (and rank) the magnitude of change in the reach, adoption, and implementation quality outcomes; and (c) to estimate how this effect differs by clinics sub-grouped on the basis of their MOUD capability at baseline, prior to enrolling in the program. It was hypothesized that (a) the primary outcome, or percent of patients prescribed MOUD, would change overtime; (b) all outcomes would improve gradually over time, with the largest magnitude of change in implementation quality, given expanding quality (or capability) is precursor to the adoption and sustainment of evidence-based practices [25]; (c) the program would have a greater effect on change over time for clinics with lower MOUD capability at baseline because of the larger opportunity for improvement.

The secondary aim estimates the effect of early engagement in the four components of the program on the outcomes of reach, adoption, and implementation quality. For each implementation strategy, the hypothesis was that early engagement in the components with a prescriber would be associated with better outcomes in future quarters than the absence of early engagement or early engagement without a prescriber. We expect that prescribers, as key actors of the implementation, must engage in the program components, in order for them to effect meaningful change [26, 27].

### Study design

**Fig. 1:**
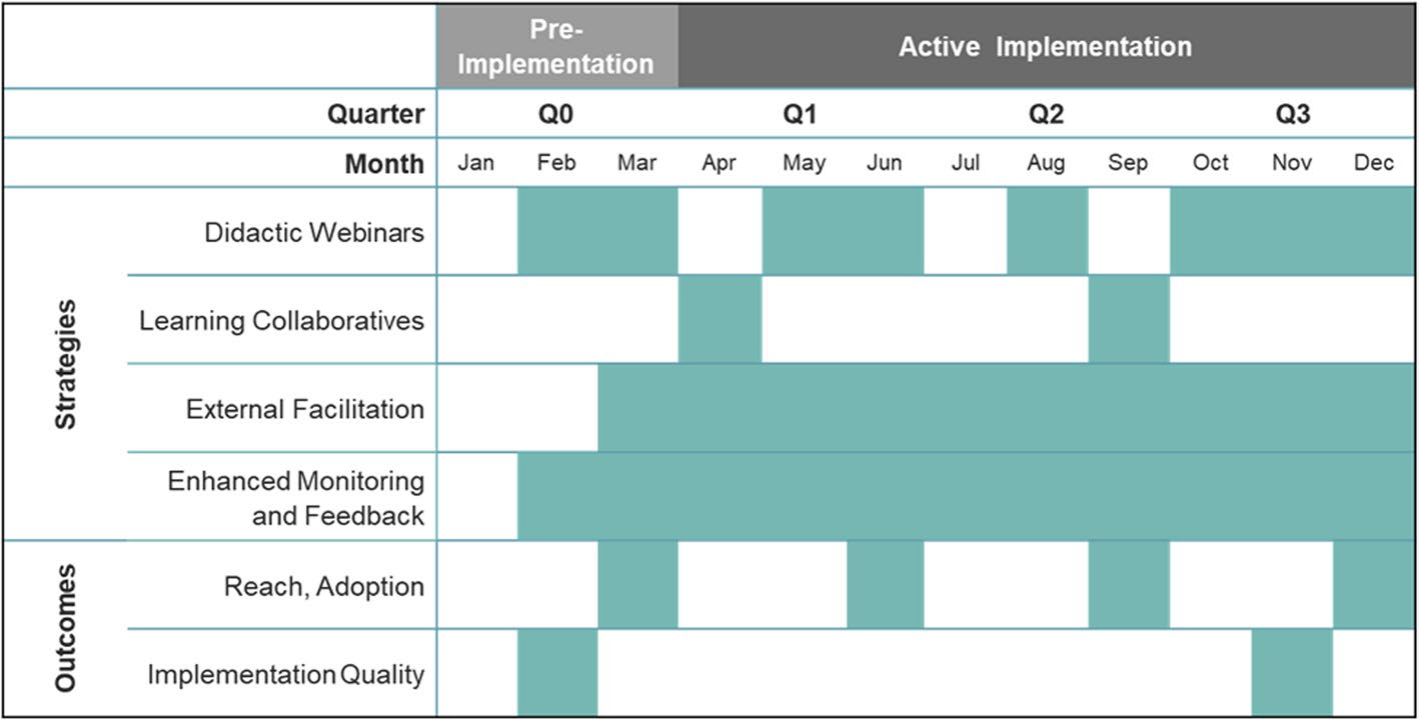
Program implementation activity and measurement timeline

This manuscript presents a prospective, longitudinal evaluation that focuses on the period from pre (January to March 2019) to mid-active implementation (October to December 2019). Figure 1 illustrates the program activities and measurement timeline. The Standards for Reporting Implementation Studies (StaRI) checklist [28] was used to guide reporting (Additional file 1).

### Clinics

Forty-two clinics (55%) out of 76 clinics that applied to participate in the program were enrolled. Clinics were eligible if they (1) provided care in the State of California; (2) met the definition of a safety-net health care organization; (3) met the definition of a non-profit and tax-exempt entity under 501(C)(3) of the Internal Revenue Service Code (IRC) or a governmental, tribal, or public entity; (4) provided comprehensive primary care services; and (5) were interested in initiating or expanding MOUD efforts. Clinics were deemed not eligible if they (1) submitted an incomplete application; (2) provided care outside of California; (3) did not meet the definition of a non-profit. Upon receipt of applications, a panel of addiction and quality improvement experts further screened potential participating clinics for MOUD program readiness, as well as team and leadership commitment. Clinics that were retained either had no existing MOUD capability (start-up clinics) or were seeking to optimize and expand any existing MOUD capability (scale-up clinics).

Clinic types included Federally Qualified Health Centers (FQHC) and FQHC look-alikes, community clinics, rural health clinics, ambulatory care clinics owned and operated by hospitals, and Indian Health Service clinics. FQHCs are nonprofits or public organizations receiving funding from the Health Resources & Services Administration and are legislatively mandated to provide primary care following a sliding fee discount program in medically underserved areas [29]. FQHC look-alikes are community health centers that provide primary care and are eligible for FQHC reimbursement structures and discounted drug pricing [30]. These clinic locations ranged from densely urban to frontier rural and primarily serve low-income patients, those living in medically underserved areas, and/or those within specific racial and ethnic subgroups (e.g., Native American Tribes and Alaska Native people, Latinos, Koreans).

**Fig. 2:**
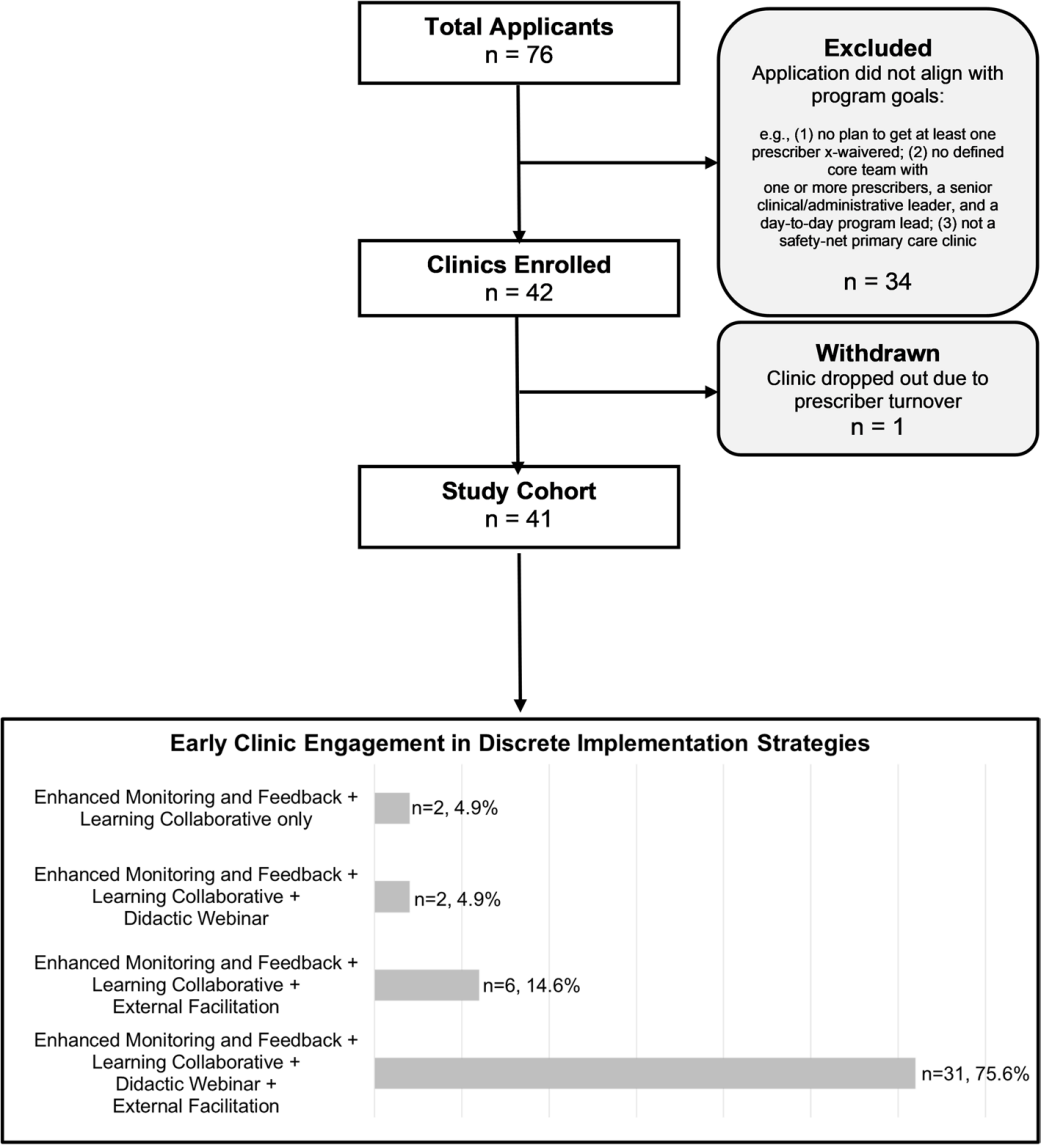
CONSORT diagram of clinic recruitment, enrollment, retention, and early engagement. Note: Under *Early Clinic Engagement in Discrete Implementation Strategies*, *n* refers to the sum of clinics that attended the first session of the specified combinations of implementation activities. Given that External Facilitation sessions are scheduled by the external facilitator and clinic team, attendance at kick-off session is only counted if it took place within the first month of active implementation

**Fig. 3:**
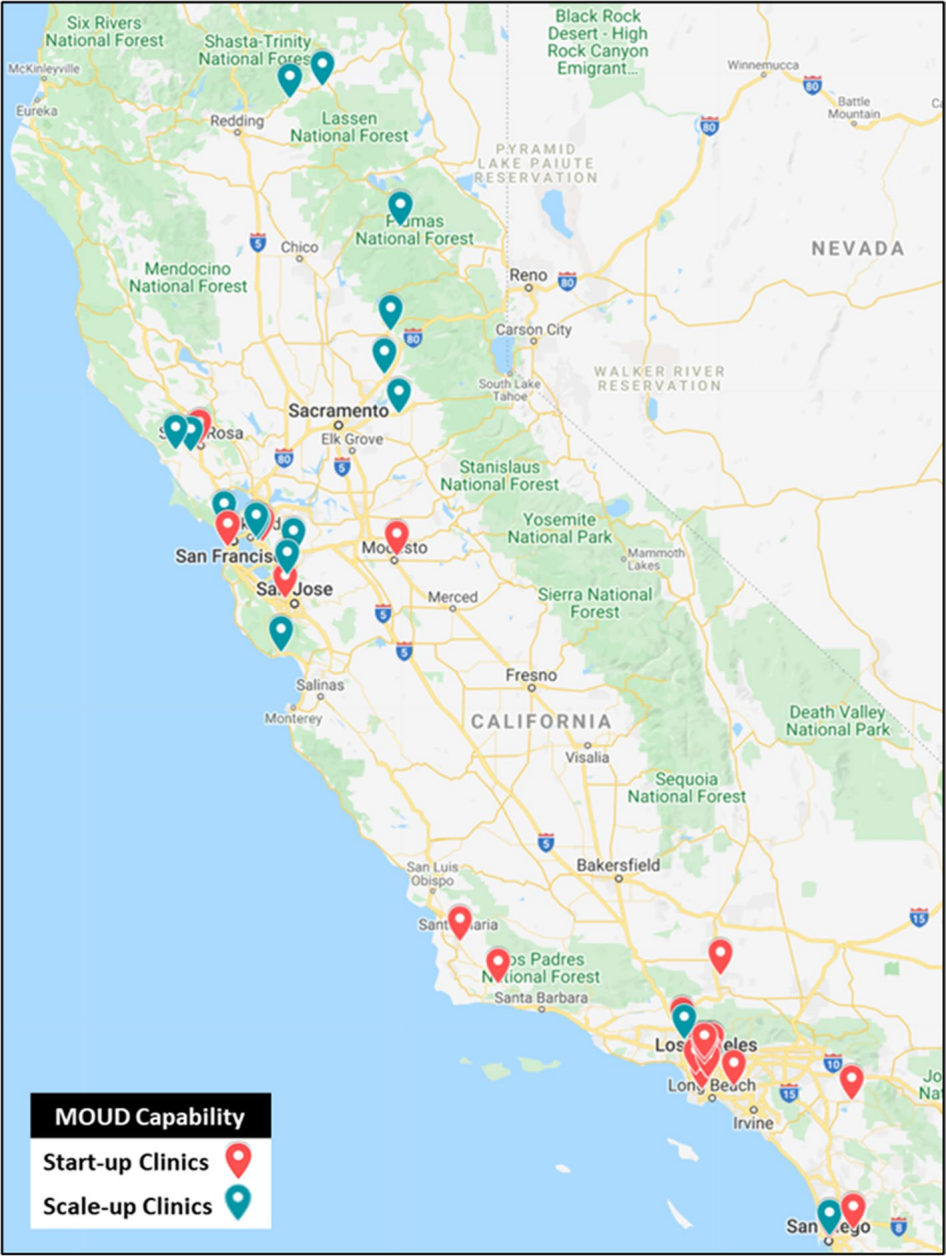
Geographic dispersion of clinic sites by baseline MOUD capability

Clinics were compensated in a stage-wise fashion with funds up to $50,000 upon meeting pre-determined benchmarks. It was made clear to all clinics and clinic leadership that this compensation was for providing research data but not for receiving or taking part in the implementation strategies. Thus, no compensation was provided to engage clinics in the implementation strategies. Following inclusion in the study, one clinic withdrew from the study. The final sample size is 41 clinics. Figure 2 presents an extended CONSORT Diagram. Figure 3 is a geographic representation of participating clinics by start-up and scale-up designation.

### Implementation strategies

Clinics were offered four types of discrete implementation support activities: (1) ongoing Enhanced Monitoring and Feedback, (2) two Learning Collaboratives, (3) monthly External Facilitation, and (4) monthly Didactic Webinars [22]. Within the confines of each strategy, the content of the implementation support activities—as well as approaches to engaging clinics in these activities—was tailored to address the barriers that patients at safety-net primary care clinics often face. However, the choice of implementation strategy offered was not tailored; instead, all four strategies were provided to all clinics as a single package (Fig. 1).

#### Enhanced Monitoring and Feedback 

Similar to audit and feedback, a commonly used evidence-based implementation strategy [31], Enhanced Monitoring and Feedback (EMF) triggers change using performance data to guide decisions and actions of quality improvement efforts [32, 33]. EMF augments traditional audit and feedback by feeding back information on organization (e.g., leadership, culture, workflow) and system-level (e.g., policies, community norms, network connectivity) factors in addition to individual performance. Throughout the pre and active implementation phases, performance indicators pertaining to reach, adoption, and implementation of MOUD were gathered and fed back to clinics quarterly via automatic run charts. Clinics were also provided with aggregate averages of all other clinics, allowing them to compare their performance with the program average.

#### Learning Collaboratives

The Learning Collaborative strategy is widely utilized across quality improvement efforts, particularly those targeting complex interventions that involve systems and multidisciplinary teams [34–36]. It also has a positive, demonstrated effect on both quality- and quantity-type outcomes for MOUD expansion in primary care [37]. Learning Collaboratives, led by a panel of addiction and quality improvement experts, were held in April and September 2019. These in-person sessions covered MOUD practice and quality improvement strategies. The MOUD practice component consisted of presentations by addiction treatment experts and primary care peers on various topics related to MOUD, such as how to kick-start MOUD; strategies to manage complex cases, diversion, patients with co-occurring stimulant use disorders, pregnancy; and approaches to address negative stigma, beliefs, and attitudes related to addressing addiction in primary care. The quality improvement segment consisted of interactive workshop activities in which clinics identified SMART (Specific, Measurable, Achievable, Relevant, and Time-bound) goals, drivers, and barriers to implement/expand MOUD practice, and setting measurable and achievable goals with team members to take back to their clinic.

#### External Facilitation

External Facilitation, sometimes also referred to as implementation facilitation, coaching, or mentoring, has been utilized extensively and successfully across a wide range of implementation efforts in medical and non-medical settings [38–42]. Clinics were assigned to an external facilitator for up to 25 h of implementation support. Clinics were encouraged to meet with their facilitator (via phone or teleconference) at least once per quarter to review ongoing MOUD expansion efforts, discuss successes, identify areas for improvement, and troubleshoot solutions to existing barriers, such as retention of MOUD care for homeless patients and those with transportation needs. The external facilitators were either addiction psychiatrists, nurses, social workers, or behavioral health specialists and had extensive experience providing MOUD care in primary care settings.

#### Didactic Webinars

Didactic Webinars have a demonstrated positive impact on reach and adoption of MOUD [20]. Fifteen, 1-h-long, webinars were developed and made available over the course of the pre and active implementation period. The webinars were led by addiction psychiatrists, nurses, social workers, or behavioral health specialists, who had extensive experience providing MOUD care in primary care settings [43]. Examples of topics covered were as follows: MOUD overview and management, contingency management, teleconsultation support for clinicians, peer support from those with lived experiences, and fundamentals of compassionate care. To acknowledge staff burnout, a common barrier in safety-net, primary care setting, Didactic Webinars integrated interactive activities such as guided meditations and open discussions that allowed staff to reflect on their work and experiences. Instant feedback from polls were collected at the end of each webinar to ensure the content was helpful and relevant.

### Measures

#### Time

In this study, time (*t*) is operationalized as the quarter (*Q*) of data collection: *t* = 0 refers to data collected in January to March 2019 (Q0; pre-implementation), *t* = 1 refers to April to June 2019 (Q1), *t* = 2 refers to July to September 2019 (Q2), and *t* = 3 refers to October to December 2019 (Q3; mid-active implementation).

#### Outcome variables

All outcomes of interest—reach, adoption, and implementation quality—were guided by RE-AIM, a framework developed and commonly used to study the quality, speed, and public health impact of efforts to implement evidence-based practices in real-world settings [44].

##### Reach and adoption

Reach and adoption were operationalized as counts and percentages for a given quarter. Reach was (1) the number of patients with a current diagnosis of OUD; (2) the number of patients with a current, active prescription of MOUD such as buprenorphine or naltrexone long-acting injectable; and (3) the percent of OUD patients with a current, active prescription of MOUD (primary outcome). A prescription was considered active if it covered any of the past 30 days of the reporting month.

Adoption was (1) the number of prescribers; (2) the number of x-waivered prescribers; (3) the number of active x-waivered prescribers who have prescribed MOUD in the 3-month data reporting period; and (4) the percent of x-waivered prescribers in the clinic.

Reach and adoption were collected quarterly via a secure online data portal customized for the program. To ensure data accuracy, clinics were trained on data collection through webinars and one-on-one phone sessions at the program start. Data validation was performed quarterly by a trained member from the research team to ensure completeness and accuracy as well as resolve discrepancies.

##### Implementation quality

The Integrating Medications for Addiction Treatment (IMAT) in Primary Care Index is a self-reported, team-based assessment designed to evaluate implementation quality of and capability for MOUD [45]. The IMAT includes elements of the Addiction Care Cascade and measures guideline adherence against policies, expert consensus recommendations, and best practices [46, 47]. It is comprised of 45 benchmark items across 7 domains that assess implementation quality with regard to Infrastructure, Clinic Culture and Environment, Patient Identification and Initiating Care, Care Delivery and Treatment Response, Care Coordination, Workforce, and Staff Training and Development. Each item is rated on a 5-point scale ranging from 1—Not Integrated to 3—Partially Integrated to 5—Fully Integrated. Scores for each domain as well as a total composite score can be generated. The IMAT is psychometrically acceptable with good internal consistency, concurrent validity, and predictive validity [48]. The IMAT was administered at baseline and at active implementation midpoint.

#### MOUD capability

Prior to implementation, each clinic was categorized as either a start-up clinic or a scale-up clinic. Start-up refers to clinics that had a small number of x-waivered prescribers with no or few patients on MOUD; scale-up refers to clinics that were more established in their OUD care. These categorizations were developed by an expert team with qualifications in addiction medicine, addiction psychiatry, primary care management, quality improvement, and correspond to Aarons et al. Exploration, Preparation, Implementation and Sustainment (EPIS) stages: Exploration and Preparation (start-up clinics) and Implementation and Sustainment (scale-up clinics) [25].

#### Early engagement

Early engagement is the focal predictor variable of the secondary aim. It was operationalized as clinic attendance at the first session of each implementation activity. Clinics were grouped into one of three categories: (1) no member of the clinic attended, (2) at least one member of the clinic attended, but none was a prescriber, and (3) at least one member of the clinic attended, including a prescriber. Prescribers are physicians, physician assistants, and nurse practitioners who are eligible for or already have an x-waiver.

Attendance data, including the name and role of attendees as well as the associated clinic, were collected by program staff at each activity. Learning Collaborative and Didactic Webinar attendance were recorded through sign-in documentation. External facilitators utilized facilitation logs to track monthly encounters. Engagement with EMF was not tracked as all clinics received it continuously throughout the program.

### Statistical analyses

Analyses were performed in R version 4.0.2. Additional file 2 provides additional details regarding the methods used.

#### Primary aim: pre-post differences of longitudinal outcomes given MOUD capability

A linear-mixed effects (LME) [49] model was used to examine changes in reach, adoption, and implementation quality as a function of time, here pre-post, and MOUD capability. The pre-specified LME model included fixed effects for the intercept, time (*t* = 0,1,2,3), MOUD capability at baseline (1 = scale-up, 0 = start-up), and an interaction term for time and baseline MOUD capability. The LME also included a random effect for intercept and an independent covariance structure for the residual errors. For ease of interpretability, MOUD capability was mean-centered, enabling the intercept and time fixed effects to be interpreted as the marginal mean at baseline and the marginal slope (i.e., averaged over MOUD capability), respectively. This same LME model was used for all three parts of the primary aim.

#### Secondary aim: effects of early clinic engagement

A LME model was used to examine the association between early engagement in each of the implementation strategies on reach and adoption. The pre-specified LME analysis included fixed effects for the intercept, time (*t* = 1,2,3), early engagement, and an interaction term for time and early engagement. The LME also included a random effect for intercept and an independent covariance structure for the residual errors.

For the implementation quality outcome, a standard regression model (not longitudinal) was fitted instead, given that implementation quality was only measured at pre-implementation (*t* = 0) and mid-active implementation (*t* = 3). This model included an intercept and early engagement.

To reduce the possibility of bias (in the estimated effect of early engagement on outcomes) due to common correlates of the outcomes and engagement [50, 51], all models included two types of variables as baseline covariates: (i) a theory-informed list of pre-implementation variables that were hypothesized to be associated with the outcomes, and (ii) a list of pre-implementation variables found to correlate moderately or strongly with early engagement. Additional information on the methods of covariate selections are provided in Additional file 2.

#### Effect sizes

For all estimated effects, effect sizes [52] are reported to ease interpretation of results and compare the effects across outcomes. An effect size of 0.5 (medium) or greater was considered clinically meaningful.

#### Missing data

For all variables considered, there was ≤10% missingness. To address missingness, the data were multiply imputed using a chained equations approach [53]. Twenty complete (imputed) datasets were generated. All results reported below were calculated using Rubin’s rules for combining the results of identical analyses on each of the 20 datasets [54, 55].

## Results

### Descriptive

#### Baseline clinic characteristics

Twenty-five (61%) clinics were MOUD start-ups, and 16 (39%) were scale-ups. The majority of clinics were FQHCs (*n* = 28; 68.3%) located in urban or metropolitan areas (*n* = 34; 82.9%). On average, clinics had less than 1 addiction certified physician (SD = 0.6) or psychiatrist (SD = 0.9) and only 1 mental health and addiction certified behavioral clinician on staff (SD = 1.5). At program start, a mean of 5 (41.3%) prescribers at each clinic were x-waivered (clinic range 0 to 23). Similarly, a mean of 30 (44.4%) patients diagnosed with OUD were receiving MOUD (clinic range 0 to 301). Baseline characteristics of clinics are presented in Table 1.

**Table 1:** Clinic characteristics at baseline (*N* = 41)

	***N***	**%**
MOUD capability		
Start-up	25	61.0
Scale-up	16	39.0
Rurality		
Urban/Metropolitan	34	82.9
Rural	7	17.1
Primary care shortage		
Non-medically underserved area	25	61.0
Medically underserved area	16	39.0
Clinic type		
FQHC	28	68.3
FQHC Look-Alikes	2	4.9
Ambulatory Care Clinic	7	17.1
Indian Health Service Clinic	4	9.8
Organization patient panel size		
Small (0–14,999 patients)	15	36.6
Medium (15,000–59,999 patients)	11	26.8
Large (≥60,000 patients)	15	36.6
	**Mean**	**SD**
General organization characteristics		
Unique patients	63,416.4	135,035.2
Employees	909.3	2,135.1
General clinic characteristics		
Physicians	16.3	34.2
Certified nurse practitioner	4.6	6.0
Physician assistants	1.8	2.5
Addiction certified physicians	0.4	0.6
Psychiatrists	0.8	1.1
Addiction certified psychiatrists	0.4	0.9
Mental health & addiction certified behavioral clinicians	1.1	1.5
MOUD characteristics		
Prescribers	20.4	33.8
X-waivered prescribers	4.9	4.7
Active x-waivered prescribers	3.6	8.0
X-waivered prescribers (%)	41.3	28.5
Patients with OUD	79.1	180.3
Patients prescribed MOUD	29.6	80.7
Patients prescribed MOUD (%)	44.4	33.6
Insurance type		
Patients on Medicaid (%)	59	19
Patients on Medicare (%)	12	17
Patients with dual eligibility (%)	7	9
Patients on private insurance (%)	8	12
Uninsured patients (%)	16	12

#### Early engagement

**Fig. 4:**
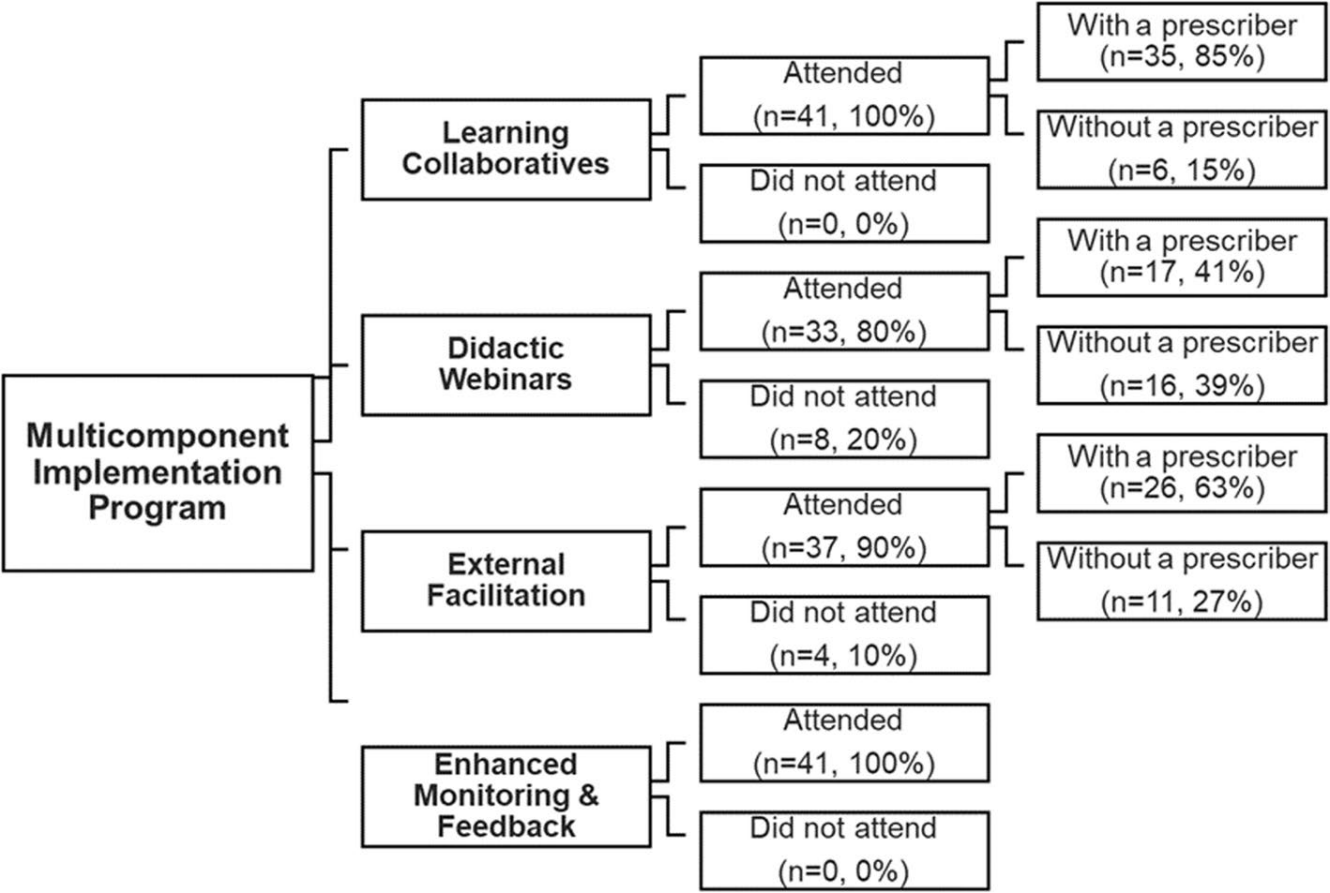
Early engagement in the multi-component implementation program (*n* = 41). Note*:* The *n*’s above represent clinic count, not individual attendees. Early engagement was operationalized as clinic attended with a prescriber, clinic attended without a prescriber, and clinic did not attend. Early engagement among providers was not reported for Enhanced Monitoring and Feedback (EMF) because all clinics received EMF continuously

Figure 4 displays a breakdown of early clinic and prescriber engagement in each of the discrete implementation strategies. All clinics attended the kick-off Learning Collaborative (*n* = 41, 100%), 37 (90.2%) clinics engaged in External Facilitation within the first 2 months, and 33 (80.5%) of clinics participated in the initial Didactic Webinar. Early prescriber engagement was the highest for Learning Collaboratives (*n* = 35, 85.4%), followed by External Facilitation (*n* = 26, 63.4%), and Didactic Webinars (*n* = 17, 41.5%).

### Primary aim: pre-post differences of longitudinal outcomes given MOUD capability

Consistent with our hypothesis, we observed pre-post difference in the percent of patients prescribed MOUD, the primary outcome (*β*_time_ = 3.99; 0.73 to 7.26; *p* = 0.02).

As anticipated, reach, adoption, and implementation quality all improved from pre to mid-active implementation (see Table 2). Implementation quality had the largest magnitude of change (ES = 0.68; 95% CI = 0.66 to 0.70), with an estimated mean improvement of 0.53 between pre to mid-active implementation. The number of x-waivered prescribers actively prescribing grew by a mean of 1.32 with the second-largest magnitude of change (ES = 0.34; 95% CI = 0.32 to 0.36). Percent of patients prescribed MOUD had an estimated mean improvement of 11.98% and the third-largest magnitude of change (ES = 0.27; 95% CI = 0.18 to 0.37).

**Table 2:** Estimated effect (EE) and effect size (ES) of change in longitudinal outcomes between program baseline (Q0) and active implementation (Q3)

	Overall	Start-up clinics	Scale-up clinics	Scale-up vs. start-up
**Reach**				
**Number of patients with OUD**				
EE (95% CI)	25.02 (−296.90, 346.94)	10.84 (−516.00, 537.68)	47.21 (−777.98, 872.40)	36.37 (−1314.97, 1387.72)
ES (95% CI)	0.11 (−1.34, 1.56)	0.05 (−2.33, 2.42)	0.21 (−3.51, 3.93)	0.16 (−5.92, 6.25)
**Number patients prescribed MOUD**				
EE (95% CI)	**12.86 (5.45, 20.28)**	9.73 (−2.42, 21.89)	17.76 (−1.25, 36.77)	8.02 (−23.14, 39.19)
ES (95% CI)	**0.19 (0.08, 0.31)**	0.15 (−0.04, 0.33)	0.27 (−0.02, 0.55)	0.12 (−0.35, 0.59)
**Percent of patients prescribed MOUD**^**a**^				
EE (95% CI)	**11.98 (7.76, 16.20)**	**22.60 (16.05, 29.15)**	**−4.63 (−7.87, −1.38)**	**−27.23 (−34.51, −19.94)**
ES (95% CI)	**0.27 (0.18, 0.37)**	**0.51 (0.37, 0.66)**	**−0.11 (−0.18, −0.03)**	**−0.62 (−0.78, −0.45)**
**Adoption**				
**Number of prescribers**				
EE (95% CI)	**1.24 (0.90, 1.59)**	**2.18 (1.61, 2.75)**	−0.23 (−1.12, 0.67)	**−2.41 (−3.87, −0.95)**
ES (95% CI)	**0.04 (0.03, 0.04)**	**0.06 (0.05, 0.08)**	−0.01 (−0.03, 0.02)	**−0.07 (−0.11, −0.03)**
**Number of x-waivered prescribers**				
EE (95% CI)	**1.40 (1.23, 1.56)**	**1.55 (1.28, 1.82)**	**1.16 (0.74, 1.59)**	−0.39 (−1.08, 0.31)
ES (95% CI)	**0.22 (0.20, 0.25)**	**0.25 (0.21, 0.30)**	**0.19 (0.12, 0.26)**	−0.06 (−0.18, 0.05)
**Number of active x-waivered prescribers**				
EE (95% CI)	**1.32 (1.25, 1.40)**	**1.54 (1.42, 1.66)**	**0.99 (0.81, 1.18)**	**−0.54 (−0.85, −0.24)**
ES (95% CI)	**0.34 (0.32, 0.36)**	**0.40 (0.37, 0.43)**	**0.26 (0.21, 0.31)**	**−0.14 (−0.22, −0.06)**
**Percent of x-waivered prescribers**				
EE (95% CI)	**3.52 (3.43, 3.61)**	**2.41 (2.27, 2.56)**	**5.25 (5.02, 5.48)**	**2.84 (2.46, 3.21)**
ES (95% CI)	**0.10 (0.10, 0.10)**	**0.07 (0.07, 0.07)**	**0.15 (0.14, 0.16)**	**0.08 (0.07, 0.09)**
**Implementation quality**				
**IMAT Implementation quality**				
EE (95% CI)	**0.53 (0.52, 0.55)**	**0.59 (0.56, 0.61)**	**0.45 (0.41, 0.49)**	**−0.14 (−0.20, −0.07)**
ES (95% CI)	**0.68 (0.66, 0.70)**	**0.75 (0.72, 0.78)**	**0.58 (0.53, 0.63)**	**−0.18 (−0.26, −0.09)**

^a^Primary outcome; EE and ES in bold indicated 95% CI did not cross zero

**Fig. 5:**
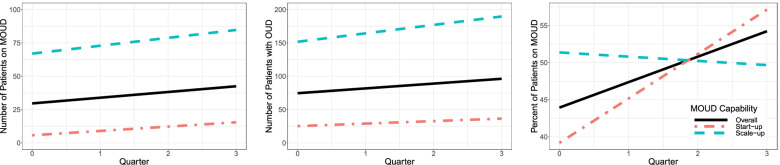
Estimated effect for reach outcomes, overall and by MOUD capability

**Fig. 6:**
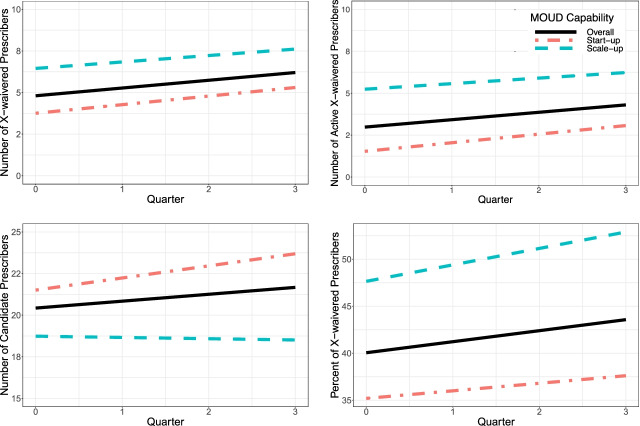
Estimated effect for adoption outcomes, overall and by MOUD capability

**Fig. 7:**
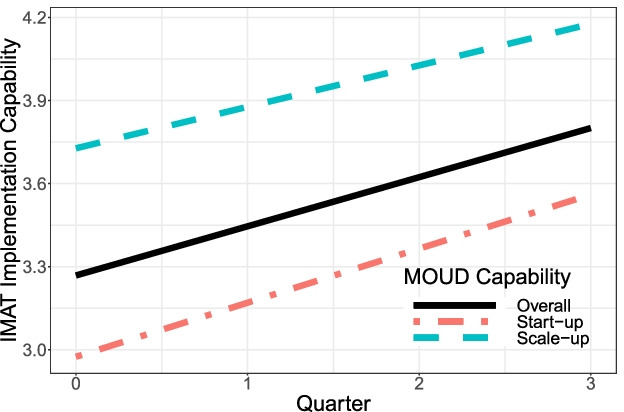
Estimated effect for implementation quality, overall and by MOUD capability

As expected, MOUD capability was found to moderate the change in longitudinal outcomes. Compared to scale-up clinics, start-ups experienced a greater, positive change in percent of patients prescribed MOUD (22.60%; 95% CI = 16.05 to 29.15), implementation quality, number of active x-waivered prescribers, and number of prescribers. In contrast (and surprisingly), scale-up clinics saw a 4.63% (95% CI = −7.87 to −1.38) reduction in the proportion of patients prescribed MOUD. Table 2 and Figs. 5, 6, and 7 respectively summarize and contrast the estimated change in longitudinal outcomes on reach, adoption, and implementation between baseline and mid-active implementation moderated by MOUD capability.

### Secondary aim: effects of early clinic engagement

**Fig. 8:**
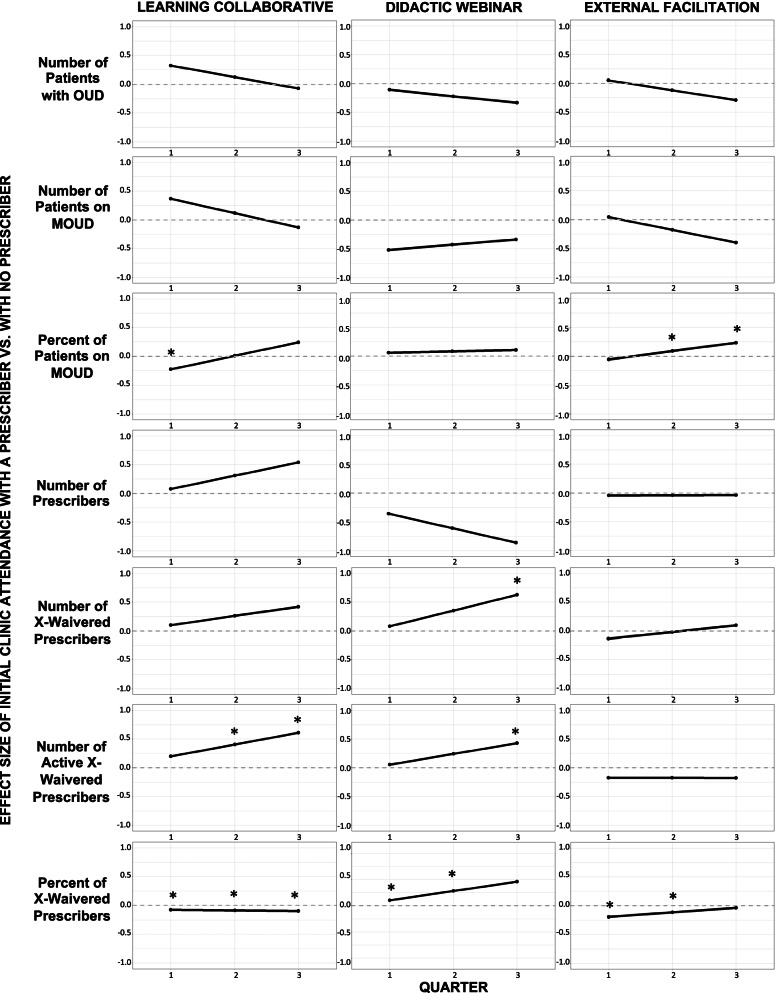
Effect size of early engagement with a prescriber compared to early engagement without a prescriber in discrete implementation strategies. Note: Asterisk (*) represents 95% CI of the effect size crossed zero; exact numerical value can be found in Tables 3, 4, and 5. Reference group is clinic attended without a prescriber. Comparison group is clinic attended with a prescriber

Tables 3, 4, and 5 present the estimated effect and effect size of clinic engagement in each implementation strategy. Figure 8 illustrates the effect size of early engagement between clinics with prescriber attendance and clinics without prescriber attendance by implementation strategy.

**Table 3:** Estimated effect (EE) and effect size (ES) of early engagement in Learning Collaboratives

	Early engagement with a prescriber vs. Early engagement without a prescriber		
	Q1	Q2	Q3
**Reach**			
**Number of patients with OUD**			
EE (95% CI)	22.77 (−1441.20, 1486.74)	8.82 (−696.67, 714.31)	−5.13 (−1468.98, 1458.72)
ES (95% CI)	0.32 (−20.34, 20.99)	0.12 (−9.83, 10.08)	−0.07 (−20.74, 20.59)
**Number of patients prescribed MOUD**			
EE (95% CI)	6.19 (−47.23, 59.60)	1.97 (−34.05, 38.00)	−2.25 (−55.66, 51.17)
ES (95% CI)	0.37 (−2.79, 3.53)	0.12 (−2.02, 2.25)	−0.13 (−3.29, 3.03)
**Percent of patients prescribed MOUD**^a^			
EE (95% CI)	**−8.02 (−12.83, −3.20)**	0.31 (−3.59, 4.22)	8.65 (1.39, 15.90)
ES (95% CI)	**−0.22 (−0.36, −0.09)**	0.01 (−0.10, 0.12)	0.24 (0.04, 0.44)
**Adoption**			
**Number of prescribers**			
EE (95% CI)	0.35 (−3.99, 4.68)	1.37 (−2.41, 5.15)	2.40 (−1.93, 6.73)
ES (95% CI)	0.08 (−0.89, 1.04)	0.31 (−0.54, 1.15)	0.54 (−0.43, 1.50)
**Number of x-waivered prescribers**			
EE (95% CI)	0.29 (−1.47, 2.05)	0.75 (−0.76, 2.26)	1.21 (−0.54, 2.97)
ES (95% CI)	0.10 (−0.51, 0.71)	0.26 (−0.26, 0.79)	0.42 (−0.19, 1.03)
**Number of active x-waivered prescribers**			
EE (95% CI)	0.36 (−0.28, 1.00)	**0.73 (0.22, 1.25)**	**1.10 (0.46, 1.74)**
ES (95% CI)	0.20 (−0.16, 0.55)	**0.40 (0.12, 0.69)**	**0.61 (0.25, 0.96)**
**Percent of x-waivered prescribers**			
EE (95% CI)	**−1.73 (−2.76, −0.70)**	**−1.94 (−2.85, −1.02)**	**−2.14 (−3.18, −1.11)**
ES (95% CI)	**−0.08 (−0.13, −0.03)**	**−0.09 (−0.13, −0.05)**	**−0.10 (−0.14 −0.05)**
**Implementation quality**			
**IMAT implementation quality**			
EE (95% CI)	–	–	**0.33 (0.25, 0.42)**
ES (95% CI)	–	–	**0.55 (0.41, 0.69)**

^a^Primary outcome; EE and ES in bold indicated 95% CI did not cross zero

**Table 4:** Estimated effect (EE) and effect size (ES) of early engagement in Didactic Webinars

	Early engagement with a prescriber vs. Did not engage			Early engagement without a prescriber vs. Did not engage			Early engagement with a prescriber vs. Early engagement without a prescriber		
	Q1	Q2	Q3	Q1	Q2	Q3	Q1	Q2	Q3
**Reach**									
**Number of patients on OUD**									
EE (95% CI)	−12.98 (−1565.22, 1539.26)	−7.63 (−702.28, 687.03)	−2.27 (−1554.45, 1549.91)	−4.21 (−1829.63, 1821.21)	10.04 (−943.41, 963.50)	24.30 (−1801.41, 1850.00)	−8.77 (−1190.90, 1173.36)	−17.67 (−635.76, 600.43)	−26.57 (−1209.28, 1156.15)
ES (95% CI)	−0.16 (−19.65, 19.33)	−0.10 (−8.82, 8.63)	−0.03 (−19.52, 19.46)	−0.05 (−22.97, 22.87)	0.13 (−11.84, 12.10)	0.31 (−22.62, 23.23)	−0.11 (−14.95, 14.73)	−0.22 (−7.98, 7.54)	−0.33 (−15.18, 14.52)
**Number of patients prescribed MOUD**									
EE (95% CI)	−1.29 (−44.71, 42.13)	2.05 (−24.66, 28.77)	5.40 (−38.03, 48.82)	6.91 (−46.69, 60.51)	8.81 (−27.74, 45.37)	10.72 (−42.88, 64.32)	−8.20 (−43.00, 26.61)	−6.76 (−30.53, 17.02)	−5.32 (−40.13, 29.48)
ES (95% CI)	−0.08 (−2.83, 2.67)	0.13 (−1.56, 1.82)	0.34 (−2.41, 3.09)	0.44 (−2.96, 3.83)	0.56 (−1.76, 2.87)	0.68 (−2.71, 4.07)	−0.52 (−2.72, 1.68)	−0.43 (−1.93, 1.08)	−0.34 (−2.54, 1.87)
**Percent of patients prescribed MOUD**^a^									
EE (95% CI)	**10.24 (0.28, 20.20)**	**−8.34 (−16.26, −0.43)**	**−26.93 (−35.23, −18.63)**	**8.15 (3.26, 13.03)**	**−11.30 (−15.46, −7.15)**	**−30.75 (−35.54, −25.96)**	2.10 (−4.88, 9.07)	2.96 (−2.04, 7.95)	3.82 (−2.63, 10.28)
ES (95% CI)	**0.28 (0.01, 0.56)**	**−0.23 (−0.45, −0.01)**	**−0.75 (−0.98, −0.52)**	**0.23 (0.09, 0.36)**	**−0.31 (−0.43, −0.20)**	**−0.85 (−0.99, −0.72)**	0.06 (−0.14, 0.25)	0.08 (−0.06, 0.22)	0.11 (−0.07, 0.29)
**Adoption**									
**Number of prescribers**									
EE (95% CI)	1.40 (−2.89, 5.69)	0.40 (−3.37, 4.17)	−0.60 (−4.89, 3.69)	2.06 (−3.34, 7.47)	1.53 (−3.34, 6.40)	1.00 (−4.41, 6.40)	−0.66 (−4.30, 2.97)	−1.13 (−4.42, 2.16)	−1.60 (−5.24, 2.04)
ES (95% CI)	0.75 (−1.56, 3.06)	0.21 (−1.81, 2.24)	−0.32 (−2.63, 1.98)	1.11 (−1.80, 4.01)	0.82 (−1.80, 3.44)	0.54 (−2.37, 3.44)	−0.36 (−2.31, 1.60)	−0.61 (−2.38, 1.16)	−0.86 (−2.82, 1.10)
**Number of x-waivered prescribers**									
EE (95% CI)	**1.45 (0.02, 2.88)**	**1.80 (0.59, 3.01)**	**2.15 (0.72, 3.58)**	1.24 (−0.62, 3.11)	0.87 (−0.77, 2.51)	0.49 (−1.37, 2.36)	0.21 (−0.91, 1.33)	0.93 (−0.04, 1.91)	**1.66 (0.54, 2.78)**
ES (95% CI)	**0.55 (0.01, 1.09)**	**0.68 (0.22, 1.14)**	**0.81 (0.27, 1.36)**	0.47 (−0.24, 1.18)	0.33 (−0.29, 0.95)	0.19 (−0.52, 0.89)	0.08 (−0.35, 0.50)	0.35 (−0.02, 0.72)	**0.63 (0.20, 1.05)**
**Number of active x-waivered prescribers**									
EE (95% CI)	0.36 (−0.26, 0.99)	0.46 (−0.05, 0.96)	0.56 (−0.07, 1.18)	0.26 (−0.55, 1.08)	0.01 (−0.68, 0.70)	−0.24 (−1.05, 0.58)	0.10 (−0.43, 0.63)	0.45 (0.00, 0.90)	**0.79 (0.26, 1.32)**
ES (95% CI)	0.20 (−0.14, 0.53)	0.25 (−0.02, 0.52)	0.30 (−0.04, 0.64)	0.14 (−0.30, 0.58)	0.01 (−0.36, 0.38)	−0.13 (−0.57, 0.31)	0.05 (−0.23, 0.34)	0.24 (0.00, 0.48)	**0.43 (0.14, 0.71)**
**Percent of x-waivered prescribers**									
EE (95% CI)	0.81 (**−**0.36, 1.97)	**5.62 (4.56, 6.68)**	**10.44 (9.27, 11.60)**	**−1.44 (−2.78, −0.09)**	**−**0.55 (**−**1.79, 0.68)	0.33 (**−**1.02, 1.67)	**2.24 (1.39, 3.09)**	**6.18 (5.39, 6.96)**	**10.11 (9.26, 10.96)**
ES (95% CI)	0.04 (**−**0.02, 0.09)	**0.26 (0.21, 0.30)**	**0.47 (0.42, 0.53)**	**−0.07 (−0.13, −0.00)**	**−**0.03 (**−**0.08, 0.03)	0.02 (**−**0.05, 0.08)	**0.10 (0.06, 0.14)**	**0.28 (0.25, 0.32)**	**0.46 (0.42, 0.50)**
**Implementation**									
**IMAT implementation quality**									
EE (95% CI)	–	–	0.05 (**−**0.04, 0.14)	–	–	**−**0.09 (**−**0.21, 0.03)	–	–	**0.14 (0.06, 0.22)**
ES (95% CI)	–	–	0.08 (**−**0.06, 0.23)	–	–	**−**0.15 (**−**0.35, 0.05)	–	–	**0.23 (0.10, 0.36)**

^a^Primary outcome; EE and ES in bold indicated 95% CI did not cross zero

**Table 5:** Estimated effect (EE) and effect size (ES) of early engagement in External Facilitation

	Early engagement with a prescriber vs. Did not engage			Early engagement without a prescriber vs. Did not engage			Early engagement with a prescriber vs. Early engagement without a prescriber		
	Q1	Q2	Q3	Q1	Q2	Q3	Q1	Q2	Q3
**Reach**									
**Number of patients with OUD**									
EE (95% CI)	17.32 (**−**2726.97, 2761.61)	6.72 (**−**1381.52, 1394.96)	**−**3.88 (**−**2748.17, 2740.41)	13.02 (**−**3292.76, 3318.81)	16.54 (**−**1687.17, 1720.25)	20.05 (**−**3286.25, 3326.35)	4.30 (**−**1234.21, 1242.81)	**−**9.82 (**−**640.28, 620.64)	**−**23.93 (**−**1263.53, 1215.66)
ES (95% CI)	0.21 (**−**33.05, 33.47)	0.08 (**−**16.75, 16.91)	**−**0.05 (**−**33.31, 33.22)	0.16 (**−**39.91, 40.23)	0.20 (**−**20.45, 20.85)	0.24 (**−**39.83, 40.32)	0.05 (**−**14.96, 15.06)	**−**0.12 (**−**7.76, 7.52)	**−**0.29 (**−**15.32, 14.74)
**Number of patients prescribed MOUD**									
EE (95% CI)	15.68 (**−**61.70, 93.06)	2.51 (**−**52.70, 57.72)	**−**10.66 (**−**88.04, 66.72)	14.99 (**−**80.07, 110.05)	5.38 (**−**63.47, 74.23)	**−**4.24 (**−**99.29, 90.82)	0.69 (**−**35.35, 36.74)	**−**2.87 (**−**28.97, 23.23)	**−**6.43 (**−**42.47, 29.62)
ES (95% CI)	0.98 (**−**3.84, 5.79)	0.16 (**−**3.28, 3.59)	**−**0.66 (**−**5.48, 4.15)	0.93 (**−**4.98, 6.85)	0.34 (**−**3.95, 4.62)	**−**0.26 (**−**6.18, 5.65)	0.04 (**−**2.20, 2.9)	**−**0.18 (**−**1.80, 1.45)	**−**0.40 (**−**2.64, 1.84)
**Percent of patients prescribed MOUD**^a^									
EE (95% CI)	4.90 (**−**3.00, 12.79)	**−**2.34 (**−**8.90, 4.22)	**−9.58 (−17.11, −2.05)**	6.91 (**−**0.59, 14.41)	**−**5.67 (**−**11.48, 0.14)	**−18.25 (−25.22, −11.29)**	**−**2.01 (**−**6.06, 2.03)	**3.33 (0.85, 5.81)**	**8.68 (4.07, 13.28)**
ES (95% CI)	0.13 (**−**0.08, 0.35)	**−**0.06 (**−**0.24, 0.11)	**−0.26 (−0.46, −0.06)**	0.19 (**−**0.02, 0.39)	**−**0.15 (**−**0.31, 0.00)	**−0.49 (−0.68, −0.31)**	**−**0.05 (**−**0.16, 0.06)	**0.09 (0.02, 0.16)**	**0.23 (0.11, 0.40)**
**Adoption**									
**Number of prescribers**									
EE (95% CI)	2.42 (**−**6.71, 11.54)	2.42 (**−**5.84, 10.69)	2.43 (**−**6.69, 11.56)	2.61 (**−**8.39, 13.61)	2.60 (**−**7.38, 12.58)	2.59 (**−**8.41, 13.59)	**−**0.19 (**−**3.72, 3.34)	**−**0.17 (**−**3.32, 2.97)	**−**0.15 (**−**3.68, 3.38)
ES (95% CI)	0.52 (**−**1.45, 2.50)	0.53 (**−**1.264, 2.314)	0.53 (**−**1.45, 2.50)	0.57 (**−**1.82, 2.95)	0.56 (**−**1.60, 2.72)	0.56 (**−**1.821, 2.940)	**−**0.04 (**−**0.81, 0.72)	**−**0.04 (**−**0.72, 0.64)	**−**0.03 (**−**0.80, 0.93)
**Number of x-waivered prescribers**									
EE (95% CI)	0.53 (**−**2.68, 3.73)	0.14 (**−**2.70, 2.98)	**−**0.24 (**−**3.45, 2.96)	0.92 (**−**3.05, 4.90)	0.20 (**−**3.35, 3.74)	**−**0.53 (**−**4.50, 3.44)	**−**0.40 (**−**1.90, 1.11)	**−**0.06 (**−**1.40, 1.11)	0.28 (**−**1.22, 1.79)
ES (95% CI)	0.17 (**−**0.89, 1.24)	0.05 (**−**0.89, 0.99)	**−**0.08 (**−**1.14, 0.98)	0.31 (**−**1.01, 1.62)	0.07 (**−**1.11, 1.24)	**−**0.18 (**−**1.49, 1.14)	**−**0.13 (**−**0.63, 0.37)	**−**0.02 (**−**0.46, 0.43)	0.10 (**−**0.40, 0.59)
**Number of active x-waivered prescribers**									
EE (95% CI)	0.62 (**−**0.48, 1.73)	0.14 (**−**0.77, 1.05)	**−**0.34 (**−**1.44, 0.76)	0.94 (**−**0.43, 2.32)	0.47 (**−**0.68, 1.61)	**−**0.01 (**−**1.38, 1.36)	**−**0.32 (**−**0.84, 0.19)	**−**0.33 (**−**0.76, 0.10)	**−**0.33 (**−**0.85, 0.19)
ES (95% CI)	0.34 (**−**0.26, 0.94)	0.08 (**−**0.42, 0.57)	**−**0.19 (**−**0.79, 0.42)	0.51 (**−**0.23, 1.26)	0.25 (**−**0.37, 0.88)	**−**0.01 (**−**0.75, 0.74)	**−**0.18 (**−**0.46, 0.11)	**−**0.18 (**−**0.41, 0.06)	**−**0.18 (**−**0.46, 0.10)
**Percent of x-waivered prescribers**									
EE (95% CI)	1.79 (**−**0.20, 3.78)	0.52 (**−**1.31, 2.35)	**−**0.75 (**−**2.75, 1.24)	**6.08 (3.61, 8.56)**	**3.08 (0.80, 5.37)**	0.08 (**−**2.39, 2.56)	**−4.30 (−5.17, −3.42)**	**−2.57 (−3.36, −1.77)**	**−**0.84 (**−**1.71, 0.04)
ES (95% CI)	0.08 (**−**0.01, 0.17)	0.02 (**−**0.06, 0.11)	**−**0.03 (**−**0.13, 0.06)	**0.28 (0.16, 0.39)**	**0.14 (0.04, 0.24)**	0.00 (**−**0.11, 0.12)	**−0.20 (−0.24, −0.16)**	**−0.12 (−0.15, −0.08)**	**−**0.04 (**−**0.08, 0.00)
**Implementation**									
**IMAT implementation quality**									
EE (95% CI)	–	–	**−0.36 (−0.53, −0.20)**	–	–	**−**0.11 (**−**0.31, 0.10)	–	–	**−0.25 (−0.33, −0.18)**
ES (95% CI)	–	–	**−0.60 (−0.87, −0.33)**	–	–	**−**0.18 (**−**0.52, 0.16)	–	–	**−0.42 (−0.55, −0.29)**

^a^Primary outcome; EE and ES in bold indicated 95% CI did not cross

Consistent with our expectation, early clinic engagement with a prescriber was associated with better outcomes in future quarters than early clinic engagement without a prescriber. Prescriber attendance at the first Learning Collaborative was moderately associated with improvement in the number of active x-waivered prescribers (ES = 0.61; 95% CI = 0.25 to 0.96) and IMAT implementation quality (ES = 0.55; 95% CI = 0.41 to 0.69). Clinics that attended the first session of Learning Collaboratives with a prescriber had an estimated mean of 1.10 (95% CI = 0.46 to 1.74) additional x-waivered prescriber actively prescribing and an addition of 0.33 (95% CI = 0.25 to 0.42) to the IMAT implementation quality score by mid-active implementation. Prescriber attendance in the first session of Didactic Webinars was moderately associated with improvement in number of x-waivered prescribers (ES = 0.61; 95% CI = 0.20 to 1.05). An estimated mean of 1.66 (95% CI = 0.54 to −2.78) additional prescribers were x-waivered in clinics that attended the early Didactic Webinar with a prescriber than those that attended without a prescriber by mid-active implementation.

## Discussion

### Summary

This study aimed to evaluate whether and to what extent a large-scale multi-component implementation support campaign led to changes in reach, adoption, and implementation quality for MOUD. Findings show that reach, adoption, and implementation quality all improved over time following the provision of a multi-component implementation program, with the greatest change on implementation quality and the least change on number of patients with OUD. These changes differed by MOUD capability at baseline. We observed the most substantial difference in the percent of patients prescribed MOUD, which improved over time for start-ups, but worsened for scale-ups. Changes in outcomes also differed by engagement in implementation strategies. Early engagement with a prescriber in the Learning Collaborative strategy had a moderate, positive effect on number of active x-waivered prescribers and implementation quality. Similarly, early engagement with a prescriber in the Didactic Webinar strategy had a moderate, positive effect on number of x-waivered prescribers.

### Implications

These findings provide insight into the effectiveness of various implementation strategies on reach, adoption, and implementation quality given determinants of context and engagement. They start informing what outcomes to target, how to target, and who/when to target—offering more precise guidance for future real-world large-scale implementation-as-usual endeavors—a departure from the “everything but the kitchen sink” approach [56]. There has been unprecedented national investment in combatting the opioid and stimulant overdose crises—with a major focus on expanding access to life-saving medications, i.e., MOUD [9–15]. Yet there is sparse scientific understanding of the effectiveness of the implementation strategies being used to scale up access to and sustain MOUD and its guideline adherent delivery [57–60]. The current study contributes to tackling this question.

#### The importance of implementation quality

Findings suggest that the multi-component implementation program had the largest effect on implementation quality. This is significant as quality and fidelity are essential to optimizing the benefit of any evidence-based intervention. As previously argued [61, 62], enhancing quality of health care delivery has the potential to maximize return on investment by “delivering the right care at the right time.” To thoroughly evaluate the effectiveness and sustainability of an implementation program, it is therefore insufficient to only evaluate the quantity as an indicator of improvement. It is also critical to examine the quality or capability of the intervention delivery as a predictor of sustainment. Capacity cannot be maximized if there is no capability. In addition to highlighting the importance of quality, our observations may imply that change in quality comes before change in quantity, which will need to be investigated in future studies. Examining the temporality of change during an implementation program can begin to unravel questions such as whether a peak in quality might predict large magnitude of improvement in reach and adoption, and whether these improvements can be sustained over time.

#### Better accommodating the needs of scale-up sites

By mid-active implementation, start-up clinics saw gains in percent of patients prescribed MOUD, whereas scale-up clinics’ percent of patients prescribed MOUD decreased. One possible explanation to this observation is that start-up clinics had more room for improvement. Some start-up clinics were new to MOUD thus had no patient with OUD or on MOUD at program start. Scale-up clinics, on the other hand, may already have a panel of OUD patients identified waiting to receive treatment, yet some of these identified OUD patients may not be eligible for MOUD. As a result, the number of OUD patients in scale-up clinics grew faster than the number of patients prescribed MOUD. This should be further investigated at program end. An alternate explanation could be the differing needs of scale-up clinics than those of start-up clinics. Clinics that are just starting up might require a higher intensity and wider range of supports, such as all four of the strategies offered in this multi-component implementation program. Sites that are scaling up, on the other hand, might experience implementation fatigue and need lighter touch but targeted scale-up strategies [63, 64], such as train-the-trainer initiatives, infrastructure development, and quality improvement collaboratives. These strategies that build human capacity and capability of implementation and develop the culture of change among staff and leaderships might be better suited to address the barriers that sites encounter during implementation scale-up. Lastly, it is also possible that improving quality may sacrifice quantity due to competing resources and demands. Through participating in this implementation effort, scale-up clinics may have realized the need to improve their quality of MOUD services. As a result, they spent more time and resources on improving their quality of care, such as existing MOUD workflow and clinic culture, thus temporarily sacrificing their goal to increasing their reach.

#### Facilitating early engagement among key actors

As expected, early engagement with a prescriber in Didactic Webinars and Learning Collaboratives predicted improvement in percent of x-waivered prescribers and implementation quality. Consistent with the literature [65], these findings suggest the importance of engaging MOUD prescribers, or key actors of the implementation effort early on. During design of implementation strategies, special attention should be given to encourage early engagement from key actors and proactively prevent disengagement.

### Limitations

Although our implementation took place across primary care clinics in the State of California, central California was underrepresented; yet, this is where implementation of MOUD may face a different set of challenges. The use of clinic-reported data, though prone to missingness and less objective than other data sources, such as claims and uniform electronic health records, is a pragmatic and affordable method of data collection that is widely utilized [66]. Our program staff anticipated for potential errors by systematically reviewing and validating the clinic-reported data to optimize data quality. Further, although clinics were instructed to complete the implementation quality team-based assessment (IMAT) as conservatively as possible, social desirability bias may have influenced implementation quality ratings.

Operationalizing engagement as attendance is only but one way to conceptualize and operationalize engagement. We were limited by the data in hand. Given the multi-dimensions and complexity of engagement, future studies should examine (i) other approaches to engaging clinics in the implementation strategies being offered; and (ii) how best to engage clinics in research (i.e., to collect outcomes of reach, adoption, and implementation quality) to further advance the field. We also believe that in many settings where research outcomes are self-reported (as opposed to passively collected), making the distinction between engagement in the implementation strategies (something an implementor does) versus engagement in obtaining the research outcome (something a researcher does) could lead to improved implementation science.

## Conclusion

These findings suggest that providing an all-clinics-get-all-components package of implementation strategies may be both inefficient and ineffective. To meet the needs of all clinics, implementers need better guidance from implementation scientists on how to select and “prescribe” the choice of initial strategy(ies) with greater precision. Similarly, implementers could also benefit from guidance on how to monitor the success of strategies early on, and how to subsequently adapt or alter the strategy(ies) being offered depending on such measures of success or failure [67, 68]. The findings also begin to point to the importance of building packages of implementation strategies that are successful at engaging clinics [27, 69, 70], or that monitor clinics for early signs of disengagement and then course-correct if needed.

## Supplementary Information

**Additional file 1.** Standards for Reporting Implementation Studies: the StaRI checklist for completion [28].

**Additional file 2.** Supplemental technical appendix [71, 72].

## Data Availability

The datasets generated during and/or analyzed during the current study are not publicly available because data belong to the participating clinics but are available from the corresponding author on reasonable request.

## Abbreviations

CI: Confidence interval
CONSORT: Consolidated Standards of Reporting Trials
EE: Estimated effect
EMF: Enhanced monitoring and feedback
ES: Effect size
FQHC: Federally Qualified Health Center
IMAT: Integrating Medications for Addiction Treatment Index
LME: Linear mixed effects
OUD: Opioid use disorder
MOUD: Medications for opioid use disorder
