# Supplementary material for: Expanding access to medications for opioid use disorder in primary care clinics: an evaluation of common implementation strategies and outcomes
Source: Implement Sci Commun. 2022 Jul 6;3:72. doi: 10.1186/s43058-022-00306-1 (PMC9258188; doi:10.1186/s43058-022-00306-1)
Supplement: Supplementary file 2 — Additional file 2. Supplemental technical appendix [71, 72]. [file 43058_2022_306_MOESM2_ESM.docx]

## Additional File 2. Supplemental technical appendix

In this appendix, we describe the analytic methods that were used to analyze the data in greater detail.

**Missing Data Approach.**  Multiple imputations using a chained equations approach was used to replace missing values (53). Here, we describe additional details concerning the extent of missing data and the methods used.

*Missing data descriptives.* There was minimal missing data in this data set. Across all baseline covariates and primary and secondary outcomes, the two variables “number of patients with OUD” and “percent of patients on MOUD” had the highest percent of missingness (Q0 - 7.32%; Q1 - 7.32%; Q2 - 9.76%; Q3 - 9.76%). *Table A* provides the descriptive statistics of missingness across all study variables. *Table B* displays the frequency of missingness by clinic.

*Selection of predictors for multiple imputation.* First, important predictors of missingness were included in the multiple imputations. To examine predictors of missingness, univariate and multivariate logistic regressions were fitted. A significant predictor of missingness was clinic type (FQHC vs FQHC look alike vs ambulatory care clinic vs Indian Health Service clinic), with ambulatory care clinics owned and operated by hospitals being predictive of missing data in “number of patients with OUD” and “percent of patients on MOUD”. Second, we included rurality as a theory-informed variable because the study included both rural and urban clinics across California. Third, we included covariates that were going to be adjusted for in the primary or secondary aim regressions: baseline MOUD capability, baseline IMAT implementation quality, baseline number of patients on MOUD, and early engagement (dummy coded clinic attendance and prescriber attendance) in Learning Collaboratives, Didactic Webinars, and External Facilitation.

*Imputation procedure.* Multiple imputation was carried out using the R package MICE v.3.13.0. (71). Selected predictors for multiple imputations described above were included in the final model. Predictive mean matching was utilized to calculate the predicted value of missing variables. Twenty complete (imputed) datasets were generated. All results reported were calculated using Rubin’s rules for combining the results of identical analyses on each of the twenty data sets (54,55).

**Linear Mixed Effects Model Specification for Primary Aim.** The primary aim sought to examine the pre-post differences of outcomes in reach, adoption, and implementation quality given baseline MOUD capability. Part (a) tested whether the provision of the multi-component implementation program impacted change in the percent of patients prescribed MOUD (primary outcome); Part (b) estimated the magnitude of change in the reach, adoption, and implementation quality outcomes; and Part (c) calculated how this effect differed by clinics sub-grouped on the basis of their MOUD capability (i.e., start-up clinics versus scale-up clinics) at baseline, prior to enrolling in the program.

*Modeling*. All models were pre-specified prior to analysis. A linear mixed effects (LME) model was used which included a random intercept and fixed effects for the intercept, time (t=0,1,2,3), MOUD capability at baseline (1=scale-up, 0=start-up), and an interaction term for time and baseline MOUD capability was fitted for all three parts of the primary aim.

*Y_ij_ = β_0_ + β_1_ time_ij_ + β_2_ MOUD capability_i1_ + β_3_ time_ij_ MOUD capability_i1_ + b_0i_ + e_ij_*

In this LME, MOUD Capability is mean-centered so that *β_1_* can be interpreted as the average effect over time marginal over MOUD Capability in Parts (a) and (b). Note that in Part (c), we interpret the effect of the multi-component implementation program conditional on MOUD capability.

*Part (a) Primary Hypothesis Test.* The primary hypothesis test was a test of the null that there is no pre-post difference in outcomes. That is, in Part (a) we tested the null hypothesis that *H_0_: β_1_ =* 0.

*Part (b) Estimated Effect Calculations.* The estimated effect ($EE$), or average pre-post difference (Q3 vs. Q0) in outcomes, is given by:

${EE}_{pre-post}=$*β_1_ * 3*

*Part (c) Estimated Effect Calculations by MOUD Capability.* The average pre-post difference (Q3 vs. Q0) for scale-up clinics is given by:

${EE}_{pre-post, Scale up}=($*β_1_ * 3) + (β_3_ MOUD capability_i1_ * 3 * 0.39),*

whereas the average pre-post difference (Q3 vs. Q0) for start-up clinics is given by

${EE}_{pre-post, Start up}=($*β_1_ * 3) + (β_3_ MOUD capability_i1_ * 3 * 0.61),*

*Standardized Effect Size Calculations*. To aide in the interpretation of the $EE$ for Parts (b) and (c), we also reported the standardized effect size, given by $ES={EE}_{pre-post}/{SD}_{pooled}$, where ${SD}_{pooled}$ is the estimated pooled standard deviation ($SD)$ of the outcome across all available time points. Based on Cohen (52), $ES$ values equal to 0.2, 0.5 and 0.8 are considered small, moderate, and large, respectively.

*Within-Clinic Correlation for Longitudinal Outcomes.* To facilitate power/sample size calculations for future researchers, we also calculated and report the within-clinic correlation for each outcome of reach, adoption, and implementation quality using the following equation (72). The within-clinic correlations are summarized in *Table C*.

$$Within­Clinic Correlation, or Intraclass Correlation Coefficient \left( ICC \right)$$

$$=\frac{random effect variance}{random effect variance+residual effect variance}$$

**Linear Mixed Effects Model Specification for Secondary Aim.** The secondary aim sought to estimate the longitudinal effect of early engagement in the three components of the program (each modeled separately) on the outcomes of reach, adoption, and implementation quality.

*Modeling*. The following pre-specified LME model that included a random intercept and fixed effects for the intercept, time (t=0,1,2,3), early engagement, and baseline predictors of early engagement (see next section for details) was fitted to estimate the effect of early engagement in Didactic Webinars and in External Facilitation, where clinics that did not attend served as a reference group. For Learning Collaboratives, a modified LME model was fitted, where early engagement without prescriber served as a reference group since all clinics attended the first session of the implementation strategy.

*Y_ij_ = β_0_ + β_1_ time_ij_ +* *β_2_ early engagement without prescriber_i1_*

*+ β_3_ early engagement with prescriber_i1_*

*+ β_4_ time_ij_ * early engagement without prescriber_i1_*

*+ β_5_ time_ij_ * early engagement with prescriber_i1_*

*+ β_x_ baseline covariates_i1_ + b_0i_ + e_ij_*

*Selection of baseline covariates.* It is important to adjust for covariates that are jointly related to both early engagement and the outcome (that is, potential confounders of the effect of early engagement on outcomes (54,55)).

Each LME included the baseline measure of the outcome as a covariate, as this is expected to be one of the strongest predictors of the outcome.

In addition, we included baseline measures found to be associated with early engagement. We utilized a two-step approach to select these measures (note that this two-step approach did not involve analyses of the outcomes). First, we considered theory-informed baseline covariates such as MOUD capability, and baseline variables that were thought to be associated with the outcome. We then quantified the relationship between each (binary or continuous) baseline covariate and early engagement in the following way: For each binary covariate, we calculated a Chi-square test and Cramer’s V effect size; and for each continuous covariate, we calculated an ANOVA test and Cohen’s d effect size. Covariates that had a medium or large effect (Cramer’s V effect size > 0.2; Cohen’s d effect size ≥ 0.5) on early engagement were selected for inclusion in the final set. For Adoption, the strongest predictor of early engagement for each implementation strategy was also included as a covariate for the respective strategy. *Table D* presents the baseline predictors of early engagement in each implementation strategy. Baseline implementation quality was a strong predictor of early engagement in all three implementation strategies. Clinics who engaged with a prescriber tended to have lower implementation quality, be MOUD start-ups, have a lower mean number of patients on MOUD, and fewer x-waivered and active x-waivered prescribers than clinics who attended without a prescriber.

The final list of baseline predictors of early engagement included in the LME model of each implementation strategy is specified in *Table E*.

*Estimated Effect Calculations*. The following equation is an example of how the estimated difference in mean change for outcomes at each time point between the engagement groups (clinic engaged with a prescriber vs. clinic engaged without a prescriber; clinic engaged with a prescriber vs. clinic did not engage; clinic engaged without a prescriber vs. clinic did not engage) was calculated. Consider calculating the effect of engaging with a prescriber vs engaging without a prescriber on the outcome at Q3 ($\text{time}\text{ij}$ = *3)*:

$${EE}_{clinic engaged with vs. without a prescriber at Q3}=(\text{β}\text{3 }\text{+ }\text{β}\text{5 * }\text{3)}-(\text{β}\text{2 }\text{+ }\text{β}\text{4 * }\text{3)}$$

*Effect Size Calculations*. We calculated standardized effect sizes (ES) by scaling the EE by the standard deviation (pooled across all time points) as shown here:

$${ES}_{clinic engaged with vs. without a prescriber at Q3}=\frac{{EE}_{clinic engaged with vs. without a prescriber at Q3}}{{SD}_{pooled}}$$

## Table A. Descriptive statistics of missing data (N=41 clinics)

| **Category** | **Variable** | | **Missing**  **(Count)** | **Missing**  **(%)** |
| --- | --- | --- | --- | --- |
| **BASELINE CLINIC FACTORS** | MOUD capability (0=start-up; 1=scale-up) | | 0 | 0.00% |
|  | Rurality (0=urban; 1=rural) | | 0 | 0.00% |
|  | Medically underserved area (no=0; yes=1) | | 0 | 0.00% |
|  | Organizational type (type 1-4) | | 0 | 0.00% |
|  | Number of unique patients within organization | | 0 | 0.00% |
| **PREDICTOR: CLINIC ENGAGEMENT** | Early engagement in Learning Collaboratives | | 0 | 0.00% |
|  | Early engagement in Didactic Webinars | | 0 | 0.00% |
|  | Early engagement in External Facilitation | | 0 | 0.00% |
| **OUTCOMES:**  **RE-AIM OUTCOMES** | **REACH** | | | |
|  | Number of patients with OUD | Q0 | 3 | 7.32% |
|  |  | Q1 | 3 | 7.32% |
|  |  | Q2 | 4 | 9.76% |
|  |  | Q3 | 4 | 9.76% |
|  | Number of patients prescribed MOUD | Q0 | 0 | 0.00% |
|  |  | Q1 | 0 | 0.00% |
|  |  | Q2 | 0 | 0.00% |
|  |  | Q3 | 0 | 0.00% |
|  | Percent of patients prescribed MOUD of all patients with OUD (primary outcome) | Q0 | 3 | 7.32% |
|  |  | Q1 | 3 | 7.32% |
|  |  | Q2 | 4 | 9.76% |
|  |  | Q3 | 4 | 9.76% |
|  | **ADOPTION** | | | |
|  | Number of prescribers | Q0 | 0 | 0.00% |
|  |  | Q1 | 0 | 0.00% |
|  |  | Q2 | 0 | 0.00% |
|  |  | Q3 | 0 | 0.00% |
|  | Number of x-waivered prescribers | Q0 | 0 | 0.00% |
|  |  | Q1 | 0 | 0.00% |
|  |  | Q2 | 0 | 0.00% |
|  |  | Q3 | 0 | 0.00% |
|  | Number of active x-waivered prescribers | Q0 | 0 | 0.00% |
|  |  | Q1 | 0 | 0.00% |
|  |  | Q2 | 0 | 0.00% |
|  |  | Q3 | 0 | 0.00% |
|  | Percent of x-waivered prescribers of all eligible providers | Q0 | 0 | 0.00% |
|  |  | Q1 | 0 | 0.00% |
|  |  | Q2 | 0 | 0.00% |
|  |  | Q3 | 0 | 0.00% |
|  | **IMPLEMENTATION QUALITY** | | | |
|  | IMAT implementation quality | Q0 | 0 | 0.00% |
|  |  | Q3 | 0 | 0.00% |

## Table B. Frequency of missingness by clinic

| **Clinic ID** | **Number of patients with OUD** | | | | **Percent of patients prescribed MOUD** | | | | **Missingness by Clinic** |
| --- | --- | --- | --- | --- | --- | --- | --- | --- | --- |
|  | **Q0** | **Q1** | **Q2** | **Q3** | **Q0** | **Q1** | **Q2** | **Q3** | **Q0 - Q3** |
| 10 |  |  | NA | NA |  |  | NA | NA | **4** |
| 16 | NA | NA | NA | NA | NA | NA | NA | NA | **8** |
| 21 | NA | NA | NA | NA | NA | NA | NA | NA | **8** |
| 22 | NA | NA | NA | NA | NA | NA | NA | NA | **8** |
| **Missingness by Variable** | **3** | **3** | **4** | **4** | **3** | **3** | **4** | **4** | **28** |

## Table C. Within-clinic correlations of primary and secondary outcomes

| **Outcomes** | **Residual Error Variance** | **Random Effect Variance** | **Within-Clinic Correlation** |
| --- | --- | --- | --- |
| **REACH** | | | |
| Number of patients with OUD | 588.02 | 28929.90 | 0.98 |
| Number of patients prescribed MOUD | 86.18 | 3276.12 | 0.97 |
| Percent of patients prescribed MOUD | 4.33 | 5.13 | 0.54 |
| **ADOPTION** | | | |
| Number of prescribers | 4.04 | 1152.39 | 1.00 |
| Number of x-waivered prescribers | 1.92 | 22.89 | 0.92 |
| Number of active x-waivered prescribers | 0.85 | 8.63 | 0.91 |
| Percent of x-waivered prescribers | 1.04 | 6.12 | 0.86 |
| **IMPLEMENTATION** | | | |
| IMAT implementation quality | 0.16 | 0.15 | 0.48 |

## Table D. Association between baseline predictors and early engagement

| **Baseline Covariates** | **Implementation Strategies** | | | | | | |
| --- | --- | --- | --- | --- | --- | --- | --- |
|  | **Learning Collaboratives** | **Didactic**  **Webinars** | | | **External**  **Facilitation** | | |
|  | **ES 2&3^a^** | **ES 1&2^a^** | **ES 1&3^a^** | **ES 2&3^a^** | **ES 1&2^a^** | **ES 1&3^a^** | **ES 2&3^a^** |
| MOUD capability  (binary**^b^**) | 0.24 | **0.49** | **0.49** | **0.49** | 0.31 | 0.31 | 0.31 |
| Number of patients with OUD (continuous**^b^**) | 0.14 | **-1.08** | -0.36 | -0.08 | 0.76 | **1.30** | 0.63 |
| Number of patients prescribed MOUD  (continuous**^b^**) | 0.76 | -0.60 | -0.37 | 0.10 | 0.35 | **1.14** | 0.63 |
| Number of x-waivered prescribers (continuous**^b^**) | 0.15 | 0.42 | 0.20 | -0.20 | 0.62 | 0.27 | -0.23 |
| Number of active x-waivered prescribers (continuous**^b^**) | 0.66 | -0.56 | -0.27 | 0.11 | -0.24 | 0.04 | 0.28 |
| Average IMAT  (continuous**^b^**) | 0.71 | **-1.51** | -0.59 | **0.95** | -0.63 | 0.43 | **1.07** |

Note: Effect sizes in bold indicated 95% CI of the effect size did not cross zero

^a^1=Clinic did not attend, 2=Clinic attended without a prescriber, 3=Clinic attended with at least one prescriber

^b^Binary covariates were tested with Chi-square test and Cramer’s V effect size; Continuous covariates were tested with ANOVA and Cohen’s d effect size

## Table E. Baseline covariates included in the secondary aim linear mixed effects model

| **Baseline Covariates** | **Implementation Strategies** | | |
| --- | --- | --- | --- |
|  | **Learning Collaboratives** | **Didactic**  **Webinars** | **External Facilitation** |
| MOUD capability | X | X | X |
| Number of patients with OUD |  | X | X |
| Number of patients prescribed MOUD | X | X | X |
| Number of x-waivered prescribers |  |  | X |
| Number of active x-waivered prescribers | X | X |  |
| Average IMAT | X | X | X |
| Baseline outcome | X | X | X |

Note: All baseline covariates included in the secondary aim model for each implementation strategy are marked with an “X”
